# Biomedical Diagnosis of Breast Cancer Using Deep Learning and Multiple Classifiers

**DOI:** 10.3390/diagnostics12112863

**Published:** 2022-11-18

**Authors:** Ahmed A. Alsheikhy, Yahia Said, Tawfeeq Shawly, A. Khuzaim Alzahrani, Husam Lahza

**Affiliations:** 1 Department of Electrical Engineering, College of Engineering, Northern Border University, Arar 91431, Saudi Arabia; 2 Department of Electrical Engineering, Faculty of Engineering at Rabigh, King Abdulaziz University, Jeddah 21589, Saudi Arabia; 3 Department of Medical Laboratory Technology, Faculty of Applied Medical Sciences, Northern Border University, Arar 91431, Saudi Arabia; 4 Department of Information Technology, College of Computing and Information Technology, King Abdulaziz University, Jeddah 21589, Saudi Arabia

**Keywords:** breast cancer, biomedical diagnosis, CNN, fuzzy algorithm, BCIC

## Abstract

Breast cancer is considered one of the deadliest diseases in women. Due to the risk and threat it poses, the world has agreed to hold a breast cancer awareness day in October, encouraging women to perform mammogram inspections. This inspection may prevent breast-cancer-related deaths or reduce the death rate. The identification and classification of breast cancer are challenging tasks. The most commonly known procedure of breast cancer detection is performed by using mammographic images. Recently implemented algorithms suffer from generating accuracy below expectations, and their computational complexity is high. To resolve these issues, this paper proposes a fully automated biomedical diagnosis system of breast cancer using an AlexNet, a type of Convolutional Neural Network (CNN), and multiple classifiers to identify and classify breast cancer. This system utilizes a neuro-fuzzy method, a segmentation algorithm, and various classifiers to reach a higher accuracy than other systems have achieved. Numerous features are extracted to detect and categorize breast cancer. Three datasets from Kaggle were tested to validate the proposed system. The performance evaluation is performed with quantitative and qualitative accuracy, precision, recall, specificity, and F-score. In addition, a comparative assessment is performed between the proposed system and some works of literature. This assessment shows that the presented algorithm provides better classification results and outperforms other systems in all parameters. Its average accuracy is over 98.6%, while other metrics are more than 98%. This research indicates that this approach can be applied to assist doctors in diagnosing breast cancer correctly.

## 1. Introduction

Various algorithms, such as those in [1,2,3,4], have been developed and implemented to detect and categorize breast tumors. The models presented in [1,2,3,4] utilize numerous technologies and tools to accomplish a reasonable range of accuracy between 98% and 98.72%. Therefore, this research intends to increase accuracy and deliver favorable conclusions. The significant contributions of this article are:

- Examining the current state-of-the-art systems for breast cancer identification and classification to identify their vulnerabilities and how to enhance their identification and classification accuracy;
- Utilizing the Fuzzy C-Means clustering algorithm and multiple classifiers to execute the classification operation precisely and achieve high accuracy;
- Evaluating the performance of the proposed system in terms of various metrics, which are accuracy, precision, recall, specificity, and F-score. This evaluation indicates that the applied method of diagnosis and classification for breast cancer reaches over 98.84% accuracy and surpasses other state-of-the-art approaches.

Breast cancer is a cancer that occurs due to uncontrollable growth of breast tissues. It can occur in both men and women, but it is more likely in women [1]. Physicians and health organizations have nominated breast cancer as one of the deadliest diseases in women [1,2]. This disease is the primary cause of death in women [1]. It is probably the most diagnosed disease on the globe. The World Health Organization (WHO) reported that the number women dying due to cancer was roughly 627,000, and this number could increase rapidly in the coming years [1,2]. It is crucial to detect breast cancer in its early stage to prepare a treatment plan to save lives [3,4,5,6]. Ductal Carcinoma in Situ (DCIS) is the most common breast cancer type. This name was given because the cancer starts and develops inside the milk ducts; 90% of reported breast cancer cases are diagnosed with this type [2,3,4,5,6,7]. In most cases, patients feel no pain, and no symptoms appear; however, symptoms and signs can appear in advanced stages, when it could be too late to treat [3,5,6,7,8,9,10]. These symptoms can be changes in shape, size, appearance, a newly inverted nipple, breast tenderness, and itching. However, breast lumps do not always imply cancer since some lumps occur due to infections [3,4,5,6,7]. A breast cancer diagnosis can be performed through self-examination, physical examination, or a mammogram.

Any uncontrollable or abnormal development in lobules or ducts will result in breast cancer. This development tends to result in lesions that may be observed by human eyes or through mammographic screening [11,12,13,14]. Mammographic screening is the most famous technology being used in the world [15,16]. Artificial Intelligence (AI) is deployed in numerous fields and applications due to its high success rate [17,18,19]. AlexNet can extract characteristics/features from images and classify these images according to the extracted characteristics.

### 1.1. Research Problem and Motivations

Physicians and radiologists scan mammographic images manually to identify breast tumors [1,2]. This process is time-consuming and unreliable and only available in big hospitals. In addition, more recent systems achieved less than 98.7% accuracy. Hence, it is necessary to develop an automatic system to overcome these limitations. This study proposes a fully automated system to identify and classify breast cancer using mammographic images. The presented method classifies tumors as either benign or malignant according to the extracted characteristics such as area, diameter, size, and location.

This research uses the fuzzy c-means clustering algorithm and multiple classifiers to detect and classify breast cancer tumors into benign or malignant. In this research, B refers to benign, and M refers to malignant. Numerous solutions have been implemented, and their classification results need greater accuracy. 

The main contributions of this study are: proposing a reliable system with high accuracy, applying the fuzzy algorithm to increase accuracy, and saving lives by identifying breast cancer earlier. These reasons motivated this study to present a fully automated system to identify and correctly classify breast tumors. This article is organized as follows: The rest of Section 1 discussed the related work on breast cancer detection and classification. The proposed system is clarified in detail in Section 2. In Section 3, the results are presented, while Section 4 provides a discussion of the obtained results. Finally, the conclusion of the paper is presented in Section 5.

### 1.2. Related Work

D. Banumathy et al. [1] presented a method to examine the accuracy of a Deep Convolutional Neural Network (DCNN) utilizing histopathological images. A Residual Network (resNet50) tool was used to obtain the discriminative localization. In addition, a Class Activation Map (CAM) was used to detect tumors and support classification procedures in the histopathological images. This method achieved 97.11% accuracy for testing, and ResNet50 reached 94.17% accuracy for the CNN. In addition, this approach classified breast cancer as benign or malignant. Two image preprocessing techniques, Binary Image Classification (BIC) and Multiclass Image Classification (MIC), were used in this method. The authors magnified the images to different values, namely, 40×, 100×, 200×, and 400×, and tested their approach on nearly 800 mammogram images. The authors found that their method generated undesired outputs when the images were magnified to 200× and 400×, while the results were good when magnified to 40× and 100×. The average accuracy for all four groups was 97.74%, while other metrics were between 95% and 97%. The proposed system in this research utilizes the fuzzy c-mean clustering approach along with AlexNet and multiple classifiers to identify and classify breast tumors as either B or M. It uses BIC and other preprocessing tools on images to allow AlexNet to extract the required features without the need to magnify images. The presented system reached over 97.8% accuracy, and other considered performance metrics were over 98%, which is much better than the implemented method in [1]. In addition, the processing time of every input was less than 5 s. In total, 3400 images were tested, and the training was conducted on more than 7000 images.

K. Nagalakshmi and S. Suriya [2] conducted a performance analysis of breast cancer detection using the ANFIS algorithm. The authors claimed that their approach was a fully automated methodology to screen cancer regions. ANFIS stands for Adaptive Neuro Fuzzy Inference System, and it is implemented for classification purposes. It performs preprocessing, feature extraction, segmentation, and classification. This method classifies tumors as either B or M, the same as the proposed system in this study. The algorithm developed in [2] utilized a dataset from the Mammographic Image Analysis Society (MIAS) to perform the required analysis. This dataset included 322 images, while the utilized datasets in this research contain more than 10,000 images. The MIAS dataset was divided into three classes, which were 208 regular tissues, 63 benign tumors, and the rest were malignant tumors. The utilized mammographic images were 1024 × 1024 pixels in size. A total of 155 images were used for training, and 167 images were used for testing. The authors measured the performance metrics of their approach in terms of accuracy, specificity, sensitivity, detection rate, positive predictive value, negative predictive value, and disc similarity coefficient. At the same time, the presented system in this research evaluates its performance metrics in terms of accuracy, precision, recall, specificity, and F-score. The developed approach in [2] achieved 98.8% accuracy, while the proposed system in this study achieved 99.02% accuracy when applied to more than 3000 images, whereas the method in [2] performed the analysis on 322 images.

In [3], V. Ulagamuthalvi et al. implemented a method to classify breast cancer using a deep convolutional neural network (DCNN), AlexNet. AlexNet is a fully automatic approach to learning and feature extraction. This method worked on low-quality images. Three datasets from the Mammographic Image Analysis Society (MIAS), DDSM-400, and CBIS-DDSM were used to train and test the developed method. Breast tumors in these three datasets were either benign or malignant. In total, 10,300 images were included in these datasets, but the authors utilized just 400 images. The authors evaluated their approach in terms of accuracy, sensitivity, specificity, precision, and F-score. The proposed system is evaluated in this study based on the same performance metrics measured in [3]. The accuracy in [3] was 98%, while the system presented here achieves nearly 99% accuracy when applied on 3400 images, instead of the 400 images used in [3]. The proposed system uses AlexNet and the fuzzy c-means algorithm to train, validate, and test this system. Ten distinct runs are used, and ten thousand iterations are set to run the system in MATLAB.

M. M. E. Eltahir and T. M. Ahmed [4] diagnosed breast tumors according to their weights using heterogeneous datasets. This model worked on three datasets and split these sets into three sub-models. Each sub-model worked independently. The authors evaluated their approach by conducting several experiments to measure accuracy, precision, and F-score, and the average obtained accuracy for all three sub-models was 81%, which is low. In contrast, the presented system in this research utilized three datasets, and the average achieved accuracy was nearly 99%, while other considered metrics were higher than 98%. The presented system is implemented based on DCNN and validated on three different datasets with more than 10,000 images in these sets. This system was trained using 100,000 images with 10,000 iterations, and it took more than 7 h, while the testing stage took nearly 2 h for 3400 images. This system generated promising outcomes and outperformed the model in [4] in terms of accuracy, precision, and F-score, as proven by the experiments.

In [6], S. Arooj et al. presented a method to detect breast cancer using a transfer learning methodology. The authors utilized three datasets, A, B, and C; dataset A was divided into two classes, A2. Ultrasound and histopathology images were used in that research. The authors customized AlexNet and utilized it on all three datasets. Dataset 1 contained ultrasound images of three different categories, which were normal, benign, and malignant. Both B and C datasets had histopathology images of two types, benign and malignant types. The number of utilized images from all three datasets was 10,336. The authors obtained the best accuracy of 99.1% on dataset C when the number of epochs was 50. In addition, the authors showed that their model achieved high accuracy when the number of epochs increased, as found in the proposed study. In contrast, the achieved accuracy of the proposed model was 99.147%, which is better than what the authors in [6] achieved. Interested readers can refer to [6] for additional information.

R. Rajakumari and L. Kalaivani [7] developed a method to identify and classify breast cancer using deep CNN techniques. The authors utilized GoogleNet and AlexNet tools as deep convolutional neural networks. Two optimizers were used, which were Root Mean Square Propagation (RMSProp) and Stochastic Gradient Descent with Momentum (SGDM), with three different learning rates, 0.01, 0.001, and 0.0001. In addition, the authors utilized numerous statistics features such as skewness, variance, and correlation to detect and classify breast cancer. The authors claimed that their approach achieved 99% accuracy, and the running time was 4.14 min. In contrast, the proposed model in this study achieved better accuracy, 99.147%, and its processing time was less than a minute, as shown in Section 3. Moreover, two different learning rates were used, which were 0.01 and 0.0001. The experiments showed that the proposed model converged faster to reach an acceptable accuracy when using 0.01, while another rate took much time to achieve the same level of accuracy.

In [8], A. Mohiyuddin et al. presented an approach to detect and categorize breast cancer in mammographic images using a Yolov5 network tool. The authors intended to lower the false positive and negative ratios and the Matthews Correlation Coefficient (MCC). The public CBIS-DDSM dataset was utilized. The authors used only 10% of that dataset for testing purposes, while the remaining 90% was used for training and validation purposes; 300 epochs and a learning rate of 0.01 were used, and a 96.5% accuracy was achieved. In addition, the authors measured recall, specificity, and precision in their implemented model which were scored as 95%, 97%, and 96.93%, respectively. In contrast, in the proposed model, 99.147% accuracy was achieved using deep CNN and three classifiers and a recall, specificity, and precision of 99.407%, 94.871%, and 99.687%, respectively, was obtained. In addition, three different datasets were utilized, while the authors in [8] used only one dataset.

R. Karthik et al. in [20] addressed the research gap in breast cancer classification from ultrasound images using a developed model. The authors implemented a novel stacked ensemble CNN approach. A Gaussian dropout layer and a customized pooling scheme were developed and utilized. The developed model classified breast tumors as healthy, benign, or malignant, which is the same as the model proposed in this research. The authors measured their algorithm performance in accuracy, F-score, precision, and recall, while the proposed model measured the performance in accuracy, precision, recall, specificity, and F-score. The authors in [20] achieved an accuracy, F-score, precision, and recall of 92.15%, 92.21%, 92.26%, and 92.17%, respectively, for their considered performance metrics. In contrast, the proposed method in this study achieved an accuracy, precision, recall, specificity, and F-score of 99.147%, 99.687%, 99.407%, 94.871%, and 99.547%, respectively. These results show that the proposed algorithm outperforms the implemented method in [20] for all measured metrics. Moreover, the authors in [20] used ultrasound images, and the proposed model utilized mammographic images. 

## 2. Materials and Methods

### 2.1. Problem Statement

Most of the latest research papers focus on recognizing and tagging breast tumors with a higher accuracy level, and the maximum obtained accuracy was 98%. In addition, the values of other evaluated performance metrics, such as precision, specificity, and F-score, were all less than 98.2%. Moreover, the processing times, also known as the execution time, of these methods are long. A longer time is considered a drawback since the identification and analysis of breast cancer should take less time. Therefore, the authors of this study aim to build and develop a reasonable, reliable, and stable system to distinguish and categorize breast cancer accurately, appropriately, and instantly while achieving higher accuracy than what has previously been accomplished.

### 2.2. Deep Convolutional Neural Network (DCNN)

**Figure 1:**
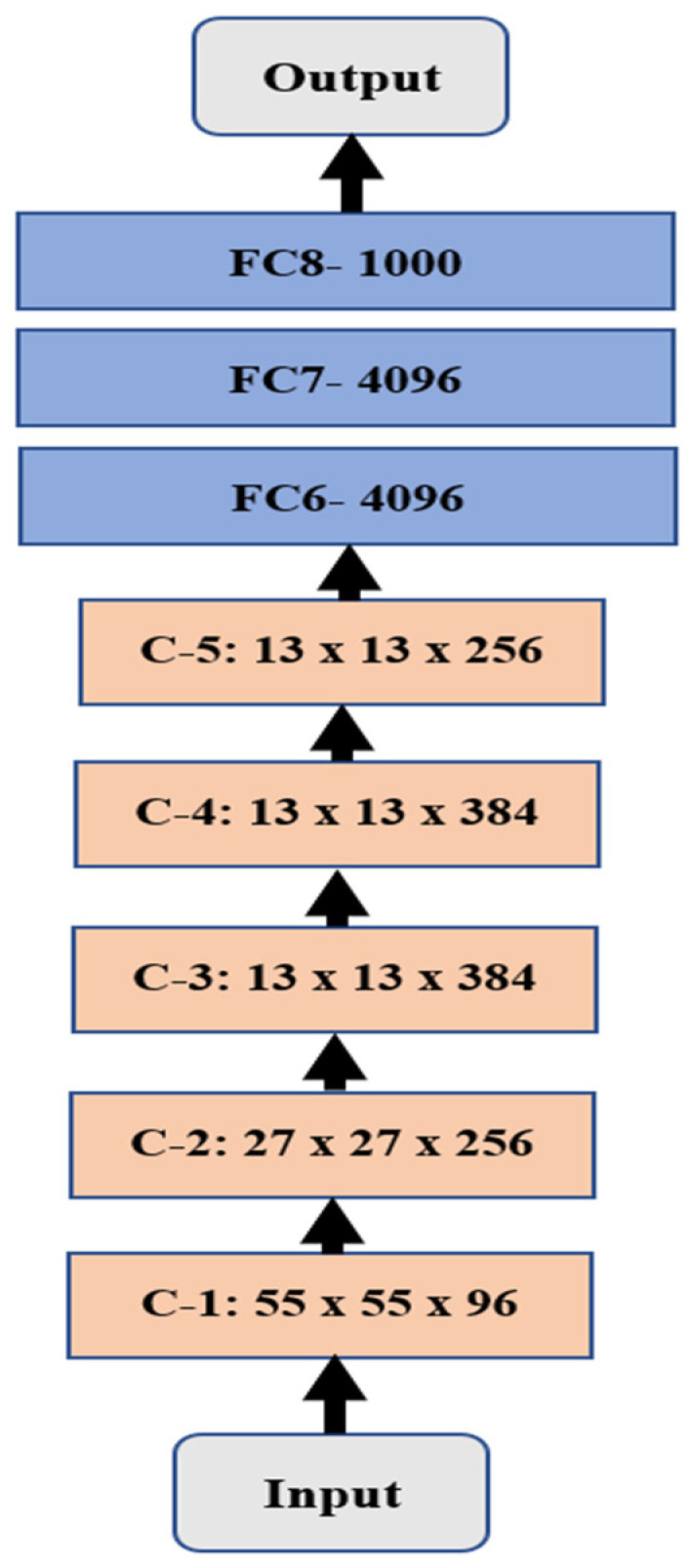
Illustration of the AlexNet structure.

A deep convolutional neural network is a type of Neural Network (NN) that is utilized in numerous Artificial Intelligence (AI) applications in the medical, industrial, and educational fields [1,3,5]. DCNN has been proven to help detect and track objects with less time and less human labor. AlexNet was implemented by two researchers in 2012 and was among the top five winners in a competition in 2012. Figure 1 illustrates the structure of AlexNet, which contains eight layers between the input and output layers. Every convolutional layer is abbreviated as C-X, where X represents the order of that layer.

The convolutional layers are distinguished in light orange with the size of every layer, while the Fully Connected (FC) layers are in light blue with the number of neurons in every layer.

### 2.3. Datasets

Three different datasets were downloaded from the Kaggle website. These datasets are Breast Histopathology Images (BHI), CBIS-DDSM Breast Images, and Breast Cancer Wisconsin (BCW). The size of the BHI dataset is roughly 3.1 GB, with more than 555,000 images; the size of CBIS-DDSM is nearly 5 GB, and the number of images is 10,237; while the BCW dataset is an Excel file with 569 entries. Every entry is associated with 31 characteristics, such as diagnosis, radius, and texture. BCW contains 357 B images and 212 M images. These three datasets are utilized in the proposed system to train and test it. In this study, 100,000 images from the BHI and DBIS-DDSM datasets were used for training, while 3400 images from both datasets were utilized for testing. The BCW dataset was used to compare the extracted features from other datasets in terms of area, diameter, radius, textures, and other features. The number of total extracted features is 18.

### 2.4. The Proposed Methodology

In this study, three various datasets are utilized to evaluate and assess the performance of the presented methodology in terms of different metrics for breast cancer identification and classification/diagnosis. These datasets contain three types of images: healthy, benign (B), and malignant (M). These are split into two categories: a training set with 100,000 images of healthy, B, and M cases and a testing set with 3400 images. The presented system ran for 7 h in training and 2 h in MATLAB testing.

**Figure 2:**
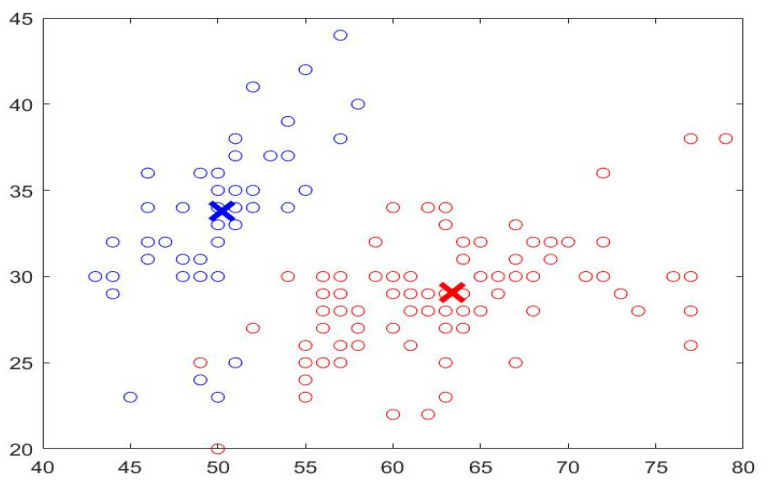
Sample of FCMC output.

This research implements a fully automated Breast Cancer Identification and Classification system (BCIC) using DCNN, the fuzzy c-mean algorithm, and multiple classifiers. The utilized classifiers are K-Nearest Neighbor (KNN), Bayes with the Gaussian kernel, and Decision Tree (DT). The fuzzy c-means clustering approach (FCMC) is used to group the potential segmented area of interest (PoI) into *n* clusters, where *n* = 2. It represents healthy and infected cells or tissues, as depicted in Figure 2. These 2 clusters are classified into their proper categories using the previous three classifiers (KNN, Bayes, and DT). In Figure 2, the blue circles refer to the nutritive values, and the red circles represent the infected cells/tissues. X represents the center of the clusters.

**Figure 3:**
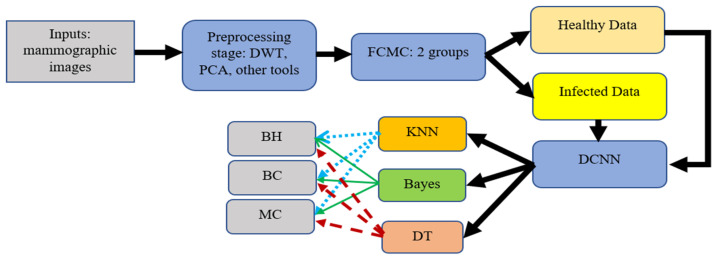
Block diagram of the proposed system.

In the preprocessing stage, various operations take place to remove noise, enhance the quality, and convert the images to gray ones. In addition, Discrete Wavelet Transformation (DWT) and Principal Component Analysis (PCA) are also used. DWT is used to denoise the inputs by removing the white Gaussian noise. PCA is utilized to reduce the obtained results to a small dimensional size. In this study, PCA reduces the dimensional size to 8 × 8. After that, the outputs are fed into the FCMC to cluster data into two groups, as depicted in Figure 2. Then, these clustered data are forwarded into the DCNN stage for the training phase. In general, FCMC is a practical algorithm for classifying data into suitable clusters. In this study, two clusters are used; these clusters are healthy data and infected data. FCMC starts by guessing a center point for each set, as shown in Figure 2; the data points close or near the blue X belong to the healthy cluster, whereas the remaining data points belong to the infected set. In this research, FCMC runs for 250 iterations to achieve better results. The training stage lasts for 7 h since 100,000 images are utilized to extract the required features such as area, diameter, texture, radius, and other features. This processing time is large; however, it is reasonable since every image is associated with 18 distinct characteristics, and in total, 1,800,000 features are generated, all of which the proposed system has to go through. Later, these extracted features are sent into the classification stage to categorize them into either healthy breast (HB), benign cancer (BC), or malignant cancer (MC). Each classifier generates its outputs, and these values are compared to determine which classifier produces exquisite findings. In addition, the obtained results are compared with other works from the literature to assess them. Figure 3 illustrates a block diagram of the proposed system.

A K-Fold Cross-Validation technique is applied to test the presented system. This procedure is a statistical method deployed to determine and evaluate the skill of the suggested model. The dataset is split into five folds since five is the minimum allowed value in MATLAB. Every fold contains the training and testing sets in different runs, as in Table 1. Every testing fold is distinguished in light blue, while the training fold is illustrated in beige.

The following pseudo-codes demonstrate how the proposed system is implemented (Algorithm 1):
**Algorithm 1**: Presented system: Breast Cancer Identification and DiagnosisInput: an image: mammographic.
Output: the detection and classification of Breast Cancer: 1) BC and 2) MC or HB.1. Read an image from a file.2. In the preprocessing phase: Do the following:3. Remove any detected noise.4. Resize the input into a compatible size with AlexNet.5. Utilize the Gabor filter, DWT, and PCA.6. Transform the resultant image into a gray image.7. End of Preprocessing phase.8. For the Deep Learning phase (DCNN): Do the following:9. Create a Zero matrix with a size = size of the input image.10. For i =1: size of the input11.     Perform a masking operation using the morphological operation to extract: Area, shape, diameter, and correlation of the potential area of Interest (PoI).12.     Determine a dynamic threshold for every image.13.     Invert the image to separate the foreground and the background.14.     Compute variance, standard deviation, mean, and correlation for every PoI in each input.15. Extract the required features.16. End17. End of DCNN phase.18. For the classification phase: Do the following:19. Create a Binary image to detect and classify the disease with a size = 1024 × 1024 in every PoI.20. Find a mass area and draw a circle around it.21. Determine the number of detected areas and their drawn circles.22. For i = 1: 102423.     For j = 1: 102424.         Compute the number of white pixels z to compare it with the threshold.25.         If z > threshold:26.         Cancer is Detected.27.     End28.     Classify detected cancer: BC or MC or display a message saying that there is no cancer.29. End30. End31. End the classification phase.32. Find TP, TN, FP, and FN.33. Compute accuracy, precision, recall, specificity, and F-score.34. End of the algorithm.


The implemented system in this study has various benefits and features, which are summarized as follows:

- The processing time is less than 13 s for every input.
- The achieved accuracy is more than 98.8%.
- The system is consistent and trustworthy.
- The system delivers favorable findings.
- The system outperforms other developed algorithms mentioned in the literature review section in all considered performance metrics.

Numerous performance metrics are computed in the developed system in this research, and these metrics are:

1. True Positive (TP): refers to the number of adequately detected and classified breast cancer images in the utilized datasets;
2. False Positive (FP): represents the number of detected and classified breast cancers classified improperly;
3. True Negative (TN): refers to the number of healthy breasts categorized accurately;
4. False Negative (FN): indicates the number of adverse outcomes classified improperly;
5. Precision (Prc): the ratio of the correctly identified types over the summation of the classes that are identified incorrectly plus the correctly identified types as demonstrated in the following equation:

Prc = TP/(TP + FP); (1)

6. Recall (Rcl): the ratio of the correctly identified classes over the summation of the actual images plus the number of antagonistic classes that are incorrectly classified, as depicted in (2) (in addition, this is sometimes called sensitivity as well):

Rcl = TP/(TP + FN); (2)

7. Accuracy (Acr): this parameter indicates how well the proposed method performs, and it is calculated as follows:

Acr = (TP + TN)/[TP + TN + FN + FP]; (3)

8. Specificity (Spc): the ability of the proposed system to classify any sample that is not associated with any labeling data. It is calculated as follows:

Spc = TN/(TN + FP); (4)

9. F-Score: the harmonic mean of the recall and precision of the implemented system. Thus, the higher the value of the F-score is, the better the model is implemented, and it is evaluated as follows:

F-Score = 2 × [(Prc × Rcl)/(Prc + Rcl)]. (5)

## 3. Results

Numerous assessment experiments were conducted in MATLAB to validate and test the proposed system’s processes and findings. This system was evaluated more than 1000 times, and it took nearly 7 h for the training phase to achieve its outcomes. In the training phase, the for-loop instruction was designed to run around 7500 times to let the system learn intensely and attain correct results. Various cases were tested to demonstrate how the system functions and acts to classify the already present breast cancer type. The calculation of the required performance metrics and a confusion matrix are presented in this section.

**Figure 4:**
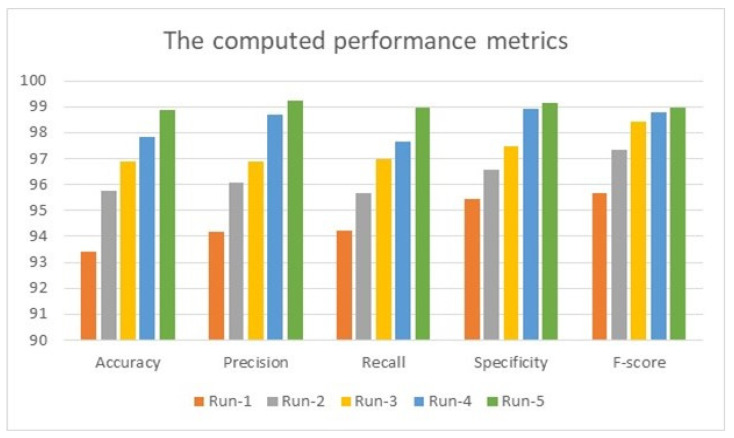
The obtained results of 5 distinct runs.

Also provided in this section is a comparative assessment between some literature works and the proposed algorithm. The use of MATLAB in all simulation scenarios was vital and dominant as it possesses built-in tools for image processing. These tools are utilized and employed in this advanced method. MATLAB is installed on a machine with Windows 11 Pro. The hosting machine contains an Intel Chip of Core i7-8550U, the clock speed is 1.8 GHz, and the RAM size is 16 GB. This machine works on a 64-bit Operating System (OS) and x64-based processor. The performance evaluation is performed and conducted in two forms, which are quantitative and qualitative, of the obtained accuracy, precision, recall, specificity, and F-score. These five metrics are essential, and various works have computed them to measure the developed algorithms. The presented system evaluated the metrics on 3400 images, and the average value was taken for every metric. This section mainly focuses on evaluating the required factors and assessing them against implemented systems of the literature review. The utilized datasets contain different sizes of images, which are resized to speed up the processing time. The training accuracy was 95.61% when running the system for just 250 iterations; this value enhanced as the number of iterations increased rapidly. For instance, the system reached 93.4%, 95.76%, 96.89%, 97.82%, and 98.9% for accuracy, precision, recall, specificity, and F-score, respectively, in the first distinct run, and these numbers improved as the number of different runs increased, as illustrated in Figure 4. The maximum obtained accuracy was 99.28%. In comparison, the F-score was nearly 99.61%, which is higher than what has been previously achieved. The chart in Figure 4 shows that all considered performance metrics increase after each distinct run and reach an acceptable level near 99% or exceed it, as the precision in run-2 is 99.24%.

Table 2 lists all values for the performance metrics calculated by the presented system. Accuracy, precision, recall, specificity, and F-score are measured in percentages. Exactly 3400 mammographic images were evaluated and tested. These images contain 1423 benign cases, 1788 malignant cases, and 189 healthy images. Furthermore, the execution time for every input was between 37 s and 57 s. These variations in processing time depend on the size of the detected mass tumor and its corresponding values of the extracted features. In addition, the number of utilized operations plays a significant role in execution, as every process takes time. All obtained results show that this system produces promising findings.

**Figure 5:**
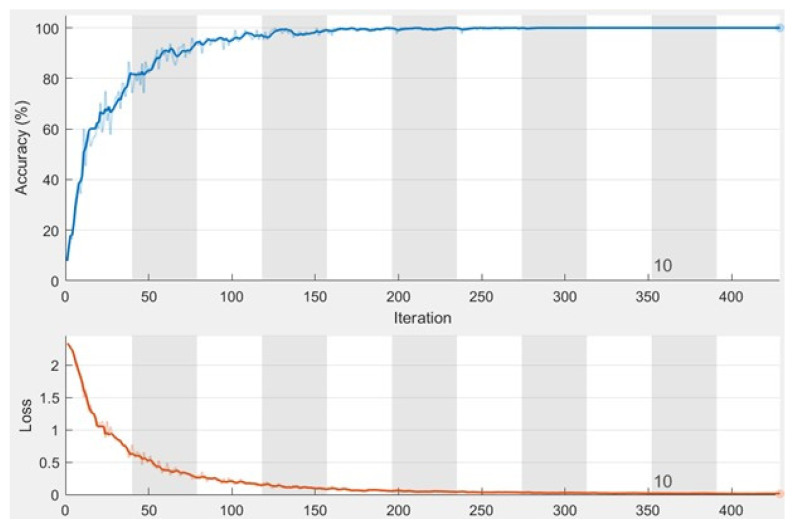
The obtained charts of accuracy and loss function.

Figure 5 shows the accuracy and the loss values when running the proposed system for 11 epochs during the training stage with a total of 429 iterations, where each epoch contains 39 iterations. In addition, the minimum batch size is 10, and the maximum batch size is 16. The number of batch sizes affects the processing time as it takes more time to process bigger batch sizes. The accuracy converges to nearly 100% after four epochs and becomes steady and stable; the same concept is applied to the loss as its value converges to 0.

Table 3 lists the accuracy classification for the testing dataset with 3400 images. The images are 2D mammographs. This set contains images of healthy breast and benign and malignant cases of cancer. This table has four columns: column 1 refers to the name of the dataset, column 2 represents the number of correctly classified images of healthy breasts (HB), column 3 represents the number of correctly classified benign cancer, and, in contrast, the last column refers to the number of classified malignant cancer.

**Figure 6:**
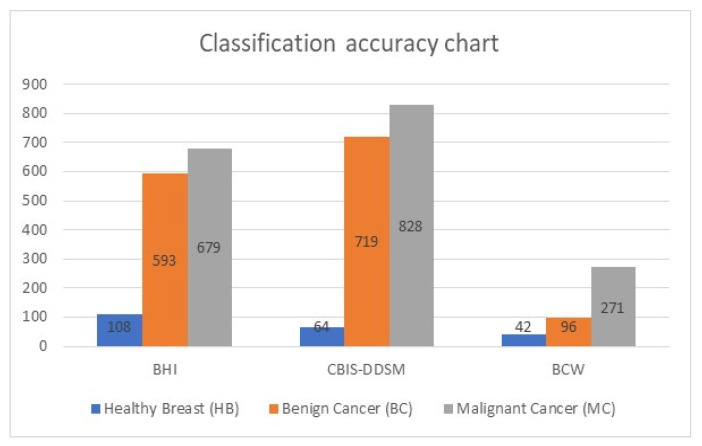
Graphical representation of the testing dataset.

Figure 6 shows the classification accuracy chart of the testing dataset. It shows the graphical representation of the total number of images and their types. In total, 29 images were classified inaccurately. 

Table 4 illustrates the confusion matrix of the obtained outputs of the implemented model. The adequately identified classes are marked in green, while the incorrectly identified classes are marked in orange.

**Figure 7:**
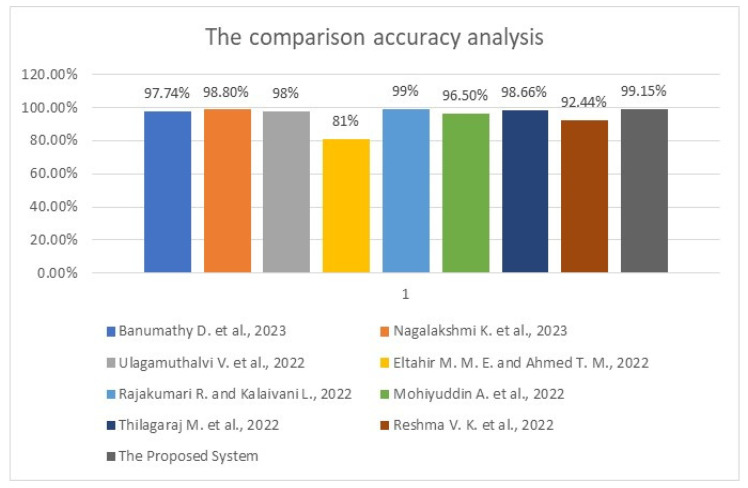
The obtained accuracy analysis charts between the proposed system and some related work [1,2,3,4] and [7,8,9,10].

The previous table shows that the developed system identified nine malignant cases, six benign cases, and three healthy cases. It detected 13 benign cases, 8 malignant cases, and 5 healthy cases, while 7 healthy cases were incorrectly categorized as 4 malignant cases and 3 benign cases. However, the system performed well and classified 3371 cases out of 3400 perfectly and precisely. Table 5 lists the assessment results for the methodology being utilized, the accuracy, the precision, the recall, the specificity, and the F-score between the proposed system and some literature works in Section 2. This table shows that the presented system in this study outperforms other works in all considered performance metrics except the recall and the specificity of [3], where the authors reached 100%. In comparison, our model achieved 99.407% and 94.871% recall and specificity, respectively. Figure 7 displays the comparative evaluation of the obtained accuracy analysis between the proposed system with recent algorithms since this metric is standard in all conducted works. This graph shows that the implemented methods in [4,10] achieved minimal accuracies of 81% and 92.44%, respectively. Furthermore, the implemented method in [8] achieved a moderate accuracy of 96.5%. Moreover, it shows that the maximum achieved accuracy before the proposed system is 99% in [7], and the presented approach in this study outperforms all methods, achieving nearly 99.2% accuracy.

## 4. Discussion

The evaluation performed on the achieved findings of the presented system shows that it detects and classifies breast cancer with higher accuracy than other developed approaches. Table 1 lists all system values when applying our system to the test dataset, while Table 3 shows how many images were detected and appropriately classified and how many images were classified improperly. In the comparative assessment between the developed system and other works from the literature shown in Table 5, the presented system outperforms others in all performance metrics, except the recall and specificity of the method in [3]. Figure 5 displays that the proposed approach achieves nearly 99% accuracy, which increases when the number of iterations and epochs increases. The maximum obtained accuracy value was 99.43%. No other method could reach this accuracy level.

The implemented system integrates the deep convolutional neural network (DCNN), AlexNet, with the fuzzy c-means algorithm and three different classifiers, KNN, Bayes, and Decision Tree, to perform deep learning and classification operations. Various phases and steps exist in this system. Integrating AlexNet and other modules eliminates confusion between the components of these stages. The resulting system accurately detects and classifies breast cancer, as shown in the previous graphs. The execution time of the developed system was 8.3 min when run for 429 iterations, meaning that the system runs at an acceptable rate for ease of use. This system reaches over 99% accuracy, outperforming studies in the literature and proving that it can be used in healthcare facilities. This system requires no special training or knowledge transfer since all operations are automated. Figure 3 illustrates how the system functions and the propagation of every output from the start to the end.

## 5. Conclusions

In this article, the proposed model demonstrates a robust and highly efficient system to detect and classify tumors from mammographic images correctly and precisely. To produce exquisite findings, this module encompasses the Gabor filter, DWT, PCA, FCMC, AlexNet, and three classifiers. It has the capability and the ability to categorize cancer as malignant or benign. The performance of the proposed system was tested on the three different datasets obtained from the Kaggle website. From the achieved outputs, it is evident that the presented method in this study outperforms all other approaches in terms of accuracy, precision, and F-score. These values show that the proposed system enhanced accuracy by 0.2020% compared with the previously highest accuracy of 99%. Overall, the proposed method offers significant improvements in considered metrics. The processing time for this system is relatively slow, as the algorithm uses various operations to obtain its high accuracy.

In terms of execution time and accuracy, improving the performance is considered for future work. The target processing time is less than 20 s for every mammographic image.

## Figures and Tables

**Table 1:** Scenario of 5-Fold Cross-Validation.

	Fold-1	Fold-2	Fold-3	Fold-4	Fold-5
Run-1	Test	Train	Train	Train	Train
Run-2	Train	Test	Train	Train	Train
Run-3	Train	Train	Test	Train	Train
Run-4	Train	Train	Train	Test	Train
Run-5	Train	Train	Train	Train	Test

**Table 2:** Evaluated performance metrics.

Performance Metric	Evaluated Values *n* = 3400
TP	3186 B = 1408, M = 1778
TN	185
FN	19
FP	10
Accuracy	99.147%
Precision	99.687%
Recall	99.407%
Specificity	94.871%
F-score	99.547%

**Table 3:** Classification accuracy of the testing dataset.

Datasets	Healthy Breast (HB)	Benign Cancer (BC)	Malignant Cancer (MC)
BHI	79	593	679
CBIS-DDSM	64	719	828
BCW	42	96	271

**Table 4:** Confusion matrix of the testing dataset.

Predicted Class.	**True Class**			
		Malignant	Benign	Healthy
	Malignant	1778 = (98.36%)	8 = (1.58%)	4 = (2.02%)
	Benign	6 = (0.98%)	1408 = (97.91%)	3 = (1.63%)
	Healthy	3 = (0.66%)	5 = (0.51%)	185 = (96.35%)

**Table 5:** The comparison evaluation results.

Works	Methodology	Accuracy	Precision	Recall	Specificity	F-Score
[1], 2023	CNN	97.74%	97.955%	95.908%	98.335%	97.863%
[2], 2023	ANFIS	98.8%	Not mentioned	97.9%	98.5%	Not mentioned
[3], 2022	DCNN	98%	99.075%	100%	100%	99.5%
[4], 2022	The weighting of heterogeneous sub-models	81%	81.14%	81.23%	Not mentioned	81.48%
[7], 2022	DCNN	99%	Not mentioned	Not mentioned	Not mentioned	Not mentioned
[8], 2022	Modified yolov5 Network	96.5%	Not mentioned	Not mentioned	Not mentioned	Not mentioned
[9], 2022	DCNN + Artificial Fish School Model	98.66%	Not mentioned	99.1%	98.8%	Not mentioned
[10], 2022	Deep Learning Models	92.44%	86.89%	Not mentioned	Not mentioned	Not mentioned
The Proposed System	DCNN + KNN + Bayes + DT	99.147%	99.687%	99.407%	94.871%	99.547%

## Data Availability

The utilized datasets in this study were downloaded from the Kaggle website, and their links are available upon request.

## References

[1] Banumathy D., Khalaf O.I., Romero C.A.T., Raja P.V., Sharma D.K. (2023). Breast calcifications and histopathological analysis on tumour detection by CNN. Comput. Syst. Sci. Eng..

[2] Nagalakshmi K., Suriya S.D. (2023). Performance Analysis of Breast Cancer Detection Method Using ANFIS Classification Approach. Comput. Syst. Sci. Eng..

[3] Ulagamuthalvi V., Kulanthaivel G., Balasundaram A., Sivaraman A.K. (2022). Breast Mammogram Analysis and Classification Using Deep Convolution Neural Network. Comput. Syst. Sci. Eng..

[4] Eltahir M.M.E., Ahmed T.M. (2022). Diagnosing Breast Cancer Accurately Based on Weighting of Heterogeneous Classification Sub-Models. Comput. Syst. Sci. Eng..

[5] Hurtado J.A.B., Albarran I.A.C., Ayala M.T., Manzano M.A.I., Hernandez L.A.M., Perez-Ramirez C.A. (2022). Diagnostic strategies for breast cancer detection: From image generation to classification strategies using artificial intelligence algorithms. Cancer.

[6] Arooj S., Rahman A.U., Zubair M., Khan M.F., Alissa K., Mosavi A. (2022). Breast Cancer Detection and Classification Empowered with Transfer Learning. Front. Public Health.

[7] Rajakumari R., Kalaivani L. (2022). Breast Cancer Detection and Classification Using Deep CNN Techniques. Intell. Autom. Soft Comput..

[8] Mohiyuddin A., Basharat A., Ghani U., Peter V., Abbas S., Bin Naeem O., Rizwan M. (2022). Breast Tumor Detection and Classification in Mammogram Images Using Modified YOLOv5 Network. Comput. Math. Methods Med..

[9] Thilagaraj M., Arunkumar N., Govindan P. (2022). Classification of Breast Cancer Images by Implementing Improved DCNN with Artificial Fish School Model. Comput. Intell. Neurosci..

[10] Reshma V.K., Arya N., Ahmad S.S., Wattar I., Mekala S., Joshi S., Krah D. (2022). Detection of Breast Cancer Using Histopathological Image Classification Dataset with Deep Learning Techniques. BioMed Res. Int..

[11] Sakib S., Yasmin N., Tanzeem A.K., Shorna F., Hasib K., Alam S.B. Breast cancer detection and classification: A comparative analysis using machine learning algorithms. Proceedings of the 3rd International Conference on Communication.

[12] Patil V., Burud S., Pawar G., Rayajaghav T. (2020). Breast cancer detection using MATLAB functions. Adv. Image Process. Pattern Recognit..

[13] Hantoro Y.N. (2020). Comparative study of breast cancer diagnosis using data mining classification. Int. J. Eng. Res. Technol..

[14] Omondiagbe D.A., Veeramani S., Sidhu A.S. (2019). Machine Learning Classification Techniques for Breast Cancer Diagnosis. IOP Conf. Series Mater. Sci. Eng..

[15] Fathy W.E., Ghoneim A.S. (2019). A deep learning approach for breast cancer mass detection. Int. J. Adv. Comput. Sci. Appl..

[16] Hamad Y.A., Simonov K., Naeem M.B. Breast cancer detection and classification using artificial neural networks. Proceedings of the 1st Annual International Conference on Information and Sciences.

[17] Tariq N. (2017). Breast Cancer Detection using Artificial Neural Networks. J. Mol. Biomarkers Diagn..

[18] Paramkusham S., Rao K.M.M., Rao B.V.V.S.N.P. Early stage detection of breast cancer using novel image processing techniques, MATLAB and Labview Implementation. Proceedings of the 15th International Conference on Advanced Computing Technologies.

[19] Cheng H.D., Shan J., Ju W., Guo Y., Zhang L. (2010). Automated breast cancer detection and classification using ultrasound images: A survey. Pattern Recognit..

[20] Karthik R., Menaka R., Kathiresan G.S., Anirudh M., Nagharjun M. (2021). Gaussian Dropout Based Stacked Ensemble CNN for Classification of Breast Tumor in Ultrasound Images. IRBM.

